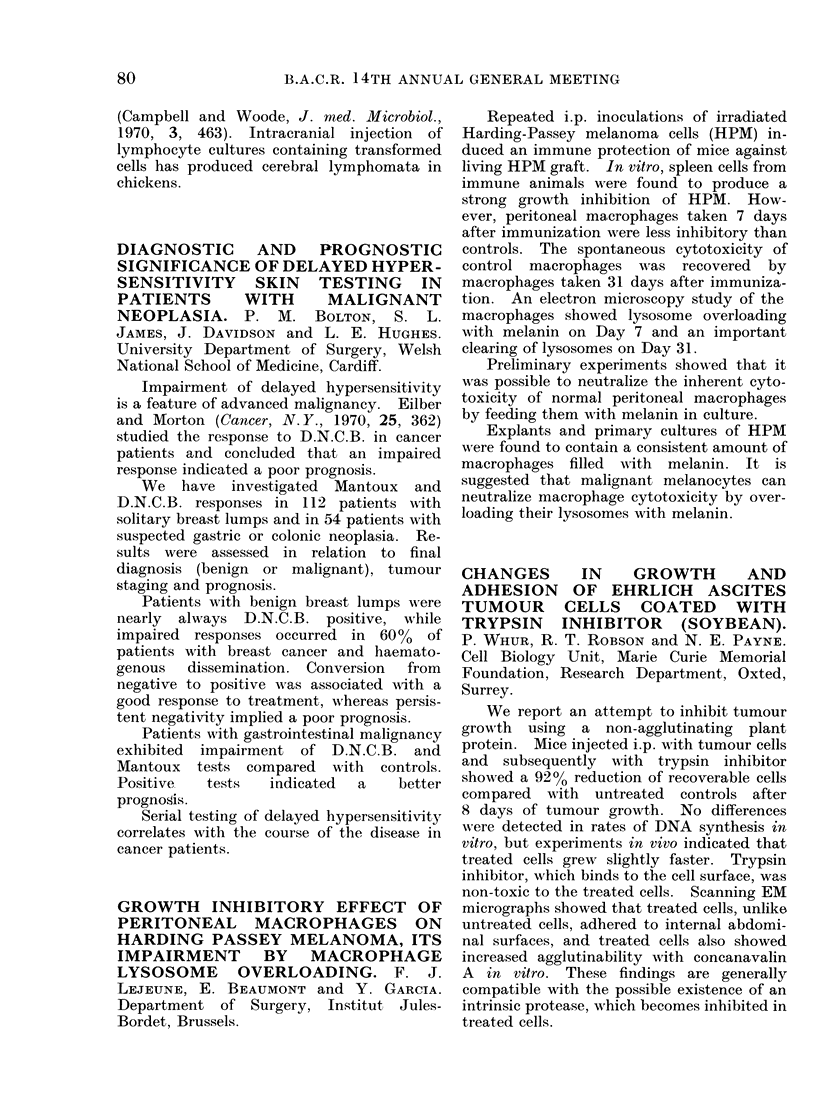# Diagnostic and prognostic significance of delayed hypersensitivity skin testing in patients with malignant neoplasia.

**DOI:** 10.1038/bjc.1973.86

**Published:** 1973-07

**Authors:** P. M. Bolton, S. L. James, J. Davidson, L. E. Hughes


**DIAGNOSTIC AND PROGNOSTIC SIGNIFICANCE OF DELAYED HYPERSENSITIVITY SKIN TESTING IN PATIENTS WITH MALIGNANT NEOPLASIA.** P. M. Bolton, S. L. James, J. Davidson and L. E. Hughes. University Department of Surgery, Welsh National School of Medicine, Cardiff.

Impairment of delayed hypersensitivity is a feature of advanced malignancy. Eilber and Morton (*Cancer, N.Y.*, 1970, **25**, 362) studied the response to D.N.C.B. in cancer patients and concluded that an impaired response indicated a poor prognosis.

We have investigated Mantoux and D.N.C.B. responses in 112 patients with solitary breast lumps and in 54 patients with suspected gastric or colonic neoplasia. Results were assessed in relation to final diagnosis (benign or malignant), tumour staging and prognosis.

Patients with benign breast lumps were nearly always D.N.C.B. positive, while impaired responses occurred in 60% of patients with breast cancer and haematogenous dissemination. Conversion from negative to positive was associated with a good response to treatment, whereas persistent negativity implied a poor prognosis.

Patients with gastrointestinal malignancy exhibited impairment of D.N.C.B. and Mantoux tests compared with controls. Positive tests indicated a better prognosis.

Serial testing of delayed hypersensitivity correlates with the course of the disease in cancer patients.